# Assessing the Feasibility of Using Parents’ Social Media Conversations to Inform Burn First Aid Interventions: Mixed Methods Study

**DOI:** 10.2196/48695

**Published:** 2024-09-26

**Authors:** Verity Bennett, Irena Spasić, Maxim Filimonov, Vigneshwaran Muralidaran, Alison Mary Kemp, Stuart Allen, William John Watkins

**Affiliations:** 1 Children’s Social Care Research and Development Centre School of Social Sciences Cardiff University Cardiff United Kingdom; 2 School of Computer Science & Informatics Cardiff University Cardiff United Kingdom; 3 Division of Population Medicine School of Medicine Cardiff University Cardiff United Kingdom; 4 School of Medicine Cardiff University Cardiff United Kingdom

**Keywords:** social media, burn first aid, health interventions, parents, burns

## Abstract

**Background:**

Burns are common childhood injuries, which can lead to serious physical and psychological outcomes. Appropriate first aid is essential in managing the pain and severity of these injuries; hence, parents who need timely access to such information often seek it from the web. In particular, social media allow them to reach other parents, hence these conversations may provide insight to aid the design and evaluation of burn first aid interventions for parents.

**Objective:**

This study aims to determine the feasibility of finding, accessing, and analyzing parent burn first aid conversations on social media to inform intervention research.

**Methods:**

The initial choice of the relevant social media was made based on the results of a parent focus group and survey. We considered Facebook (Meta Platforms, Inc), Mumsnet (Mumsnet Limited), Netmums (Aufeminin Group), Twitter (subsequently rebranded as “X”; X Corp), Reddit (Reddit, Inc), and YouTube (Google LLC). To locate the relevant data on these platforms, we collated a taxonomy of search terms and designed a search strategy. A combination of natural language processing and manual inspection was used to filter out irrelevant data. The remaining data were analyzed manually to determine the length of conversations, the number of participants, the purpose of the initial post (eg, asking for or offering advice), burn types, and distribution of relevant keywords.

**Results:**

Facebook parenting groups were not accessed due to privacy, and public influencer pages yielded scant data. No relevant data were found on Reddit. Data were collected from Mumsnet, Netmums, YouTube, and Twitter. The amount of available data varied across these platforms and through time. Sunburn was identified as a topic across all 4 platforms. Conversations on the parenting forums Mumsnet and Netmums were started predominantly to seek advice (112/116, 96.6% and 25/25, 100%, respectively). Conversely, YouTube and Twitter were used mainly to provide advice (362/328, 94.8% and 126/197, 64%, respectively). Contact burns and sunburn were the most frequent burn types discussed on Mumsnet (30/94, 32% and 23/94, 25%, respectively) and Netmums (2/25, 8% and 14/26, 56%, respectively).

**Conclusions:**

This study provides a suite of bespoke search strategies, tailored to a range of social media platforms, for the extraction and analysis of burn first aid conversation data. Our methodology provides a template for other topics not readily accessible via a specific search term or hashtag. YouTube and Twitter show potential utility in measuring advice offered before and after interventions and extending the reach of messaging. Mumsnet and Netmums present the best opportunity for informing burn first aid intervention design via an in-depth qualitative investigation into parents’ knowledge, attitudes, and behaviors.

## Introduction

### Background

Burns are a leading cause of childhood injury globally [1] and can have serious lifelong psychological [2-5] and physical health outcomes [6-9]. Appropriate and timely burn first aid (BFA) can significantly reduce the pain and severity of a burn injury [10]. However, it has been extensively documented that parents’ knowledge [11-13] and practice [14] of appropriate first aid tend to be inadequate.

Scientific evidence supports cooling the burn for 20 minutes under cool running water, calling for medical advice, and then covering the burn with a clean nonfluffy dressing [15]. Yet scientifically unsupported and traditional remedies are frequently used by caregivers, many of which may (unintentionally) cause additional harm. For example, butter, oil, turmeric, egg white [16], and even fountain pen ink [17] have been used by caregivers to treat serious children’s burns before hospital attendance despite such remedies being contrary to the advice offered by health services in high-income countries (eg, the United Kingdom, the United States, and Australia). Health service advice is published on the internet (eg, National Health Service Direct), and although parents may look up this advice directly, there is also evidence that parents turn to social media for ad hoc support [18-21].

Previous research has shown that many parents would find it more convenient to look up information on the internet over seeking health professional advice [21]. A study of millennial parents reported that almost 20% rely on the internet (eg, blogs, parenting sites, and social media) for parenting advice [22]. The relative anonymity, convenience, and availability of large communities of shared experience have been cited as particular draws for parents [19,23]. However, in the face of a high volume of conflicting, inaccurate BFA advice from the web [24-27], obfuscation of good advice and increased circulation of harmful “remedies” via social endorsement are likely dangers. Hence, effective intervention to better deliver good first aid information to parents is urgently needed.

Social media conversations present an untapped resource for both designing and measuring the short- and long-term effects of web-based safety interventions. They can offer insight into baseline knowledge and behavior and any demographic patterns (who says and does what), reach, attitudes toward campaign messages and materials (who shares what and why), and any changes in knowledge and behavior following the launch of an intervention.

To date, research into parents’ use of social media for health advice has mostly focused on the perinatal period [23,28-30], breastfeeding [31-33], and vaccinations [34-38]. We do not know to what extent parents use social media to discuss first aid for burns and if so, which platforms they use to do so, what the nature of their discussions are, and to what extent this information can be accessed and harnessed for intervention research purposes.

### This Study

This study aimed to determine the feasibility of finding, accessing, and analyzing parent BFA conversations on social media to inform intervention research.

## Methods

### Overview

Study objectives were to identify relevant social media platforms (SMPs) and then to identify and extract data from relevant conversations within these SMPs. We then assessed the utility of data accessed from each platform in terms of timescale and volume of available data, number of users, length of conversations, whether the initial post was offering or asking for advice, the type of burn injuries discussed, and frequency of first aid terms.

### Ethical Considerations

Ethical approval for this study was given by the Cardiff University School of Medicine Research Ethics Committee (approval 19/110).

Survey participants were recruited via web-based advertisements on Facebook (Meta Platforms, Inc) and Twitter (subsequently rebranded as “X”; X Corp). Before commencing the survey, participants were asked to consent to participate. The option of entering a £50 (US $62) prize draw was offered to those taking part in surveys. Parents contributed to the survey design via group discussion.

The social media conversation data accessed for this study were made publicly available by the users under the terms of service of the corresponding SMPs. These terms specifically state that users’ posts (including post content and associated metadata) are publicly accessible. Wherever possible, data were accessed by their application programming interfaces (APIs). Permission to extract data via a web scrape was sought from administrators of parenting forums for this specific research project on the basis that this was within the terms of their user agreements.

Consent was not sought from individual SMP users. Owing to the large scale of the data to be collected, it was not practical or possible to seek the consent of all users who generated the data. The lawful basis for not seeking consent or notifying users is in line with fair processing guidelines set out in General Data Protection Regulation legislation [39]. This research is deemed to fall under public task (the processing is necessary for the research team to perform a task in the public interest) as set out in Article 6 of the General Data Protection Regulation.

A Data Privacy Impact Assessment was completed in the interest of minimizing and mitigating the privacy risk involved in collecting, storing, and processing data extracted from SMPs. All data were stored on secure servers behind the University’s firewall.

### Platform Identification and Accessibility

Parents informed the study design by identifying relevant SMPs and informing the list of search terms that we used to extract user-generated data. We ran a face-to-face focus group at a children’s center in South Wales (December 2019) to inform a web-based survey (March 2020; Multimedia Appendix 1) using social media paid advertising on Twitter and Facebook to recruit parent participants from a wide geographic area across the United Kingdom.

The most popular SMPs used by parents in our focus group and the wider survey group included Facebook, Twitter, Mumsnet (Mumsnet Limited), Netmums (Aufeminin Group), Instagram (Meta Platforms, Inc), YouTube (Google LLC), and Pinterest (Pinterest, Inc). Facebook parenting groups and Instagram, alongside some less frequently used SMPs for parenting advice identified in our parent survey (eg, WhatsApp [Meta Platforms, Inc], Snapchat [Snap Inc], TikTok [ByteDance Ltd], Pinterest, and Ravelry [Ravelry, LLC]) hosted private conversations, which required an account sign in or group membership to access the content. This proved incompatible with our ethics approval and thus was not pursued.

Consequently, we investigated Mumsnet, Netmums, YouTube, Twitter, and Facebook “pages” of any influencers or organizations reported as sources of parenting advice in the survey. Reddit (Reddit, Inc) was also investigated, despite not being frequently cited in the survey, due to its popularity with men [40] to increase the chance of including fathers in our data.

### Search Strategy and Data Collection

A taxonomy of search terms (Textbox 1) was collated using the results of our parent survey and frequently occurring terms in a research database of children attending the emergency department (ED) with a burn injury [41]. Search terms were grouped into 3 categories, including burn injury, first aid (general, specific, and food-based), and references to children. The last category was used on platforms that were not specifically targeted at parents to identify conversations between parents or caregivers. In addition, to automatically filter out large amounts of irrelevant data from Twitter, a set of exclusion terms was also compiled (Textbox 1). Typical examples of irrelevant content include references to burnout as an occupational phenomenon, exercising to burn calories or energy, burning the house down either literally or figuratively, etc. Search terms were combined into queries compatible with the type of SMP and the limitations of its search capabilities.

In light of Facebook’s increasing API restrictions on developers, we resorted to using the NVivo (Lumivero) plugin NCapture [42] to access the content of influencer and organizational pages. However, this software did not provide the functionality to selectively extract content based on search terms. Instead, the full content of these pages was stored and then searched term by term within NVivo software (Version 12 plus) [43].

Discussion board areas of Mumsnet and Netmums were searched using custom search engines created in Microsoft Bing Custom Search. Due to limitations on the number of search terms in a single query, 3 queries were executed within each website, each combining “burn injury” terms with the “general,” “specific,” or “food”-related first aid terms, respectively (Textbox 1). It was assumed that all voices on these parenting forums were those of children’s parents or caregivers; hence, it was considered unnecessary to specifically search for references to children. Whole conversation threads were extracted if they contained any post that satisfied the search criteria. Results from the 3 searches were merged to remove duplicate conversations between and within the separate queries.

Initial investigation of “r/Parenting” and “r/AskParents” subreddits using search functions in Reddit API returned fewer results than using Microsoft Bing Custom Search. This appeared to be due to Reddit indexing thread titles rather than the whole thread. Therefore, we used Microsoft Bing Custom Search again to identify relevant posts. Once a relevant post has been identified, the remaining posts within the same conversation thread were then collected using Reddit API.

YouTube search and data collection were performed using YouTube APIs. Results for each query were limited to approximately 500 to 550 results due to a soft limit imposed by the YouTube search algorithm. Results varied depending on the order in which search terms were entered into the query. For example, if a query was set to request a word from each of the “burn,” “first aid,” and “child” keyword lists, the results would include frequent cases where terms from only the first 2 sets of keywords were returned. This is owing to the elimination of keywords that reduce the ranking of the results by the YouTube algorithms. These query limitations on both the number of results returned and the extent to which search results satisfy the query conditions set were not improved using Google Video Search (Google LLC) or Bing Video Search. To improve the relevance of results in the data extraction via the YouTube API, video titles, descriptions, and tags were queried for those containing terms appearing in only the “burn” and “general” first aid keyword lists. As search results were limited to approximately 500, it was possible to manually select potentially relevant videos from the results list for which to extract all associated comments.

The Twitter search and data extraction was conducted using Twitter Developer APIs. Twitter search was broken down into 3 queries because of a limit on the number of characters (1024) permitted in a query. Each query combined a “burn injury” term, a “first aid” term (limited to either “general,” “specific,” or “food item” terms for each search), and a “child” descriptor term and excluded any tweets containing an “exclusion term.”

All data were extracted between June and July 2020 in JavaScript Object Notation except for Facebook data, which were extracted as NVivo files. No date limit was imposed on search data, meaning that data time frames covered a period between the search date and the oldest post, meeting our search criteria on each platform.

**Taxonomy of search terms used to identify burn first aid conversations collated from parent survey and emergency department burns research database.**

**Burn injury**

- burn, burns, burned, burnt, scald, scalded, sunburn, sunburned, sunburnt

**First aid**

- General
  treatment, treat, remedy, cure, first aid, 1st aid, advice, medicine, heal, soothe
- Specific
  water, tap, shower, ice, frozen peas, compress, wet cloth, wet towel, aloe, moisturiser, e45, aftersun, lotion, calamine, gel, Sudocrem, Savlon, Germaline, Acriflex, cream, lotion, Vaseline, ibuprofen, aspirin, paracetamol, clingfilm
- Food
  butter, margarine, oil, honey, egg, yoghurt, potato, onion, turmeric, flour, milk

**Children**

- my kid, little one, infant, child, toddler, kiddo, youngster, year old, yo, month old, mo old, baby, babbie, babe, bub, bairn

**Twitter exclusion terms**

- out, down, calories, calorie, energy, hell, bridges, ground, house

### Data Filtering

Once downloaded, the data were filtered to reduce the amount of irrelevant content. The title and main post were analyzed for Netmums, Mumsnet, and Reddit. The video title and comments were analyzed for YouTube. For Twitter, the tweet itself and extended text fields were all considered. Any emoticons, symbols and pictographs, transport symbols, map symbols, other emojis, and punctuation were removed before text analysis. Stop words such as “the,” “is,” or “was” were removed, and the remaining words were lemmatized, that is, replaced by their dictionary form.

Some of the search terms used to identify BFA conversations (Textbox 1) are ambiguous. For example, in addition to thermal injury, “burn” can also mean to expend energy (burn calories), run out of energy (burnout), be slang for insult, or be used figuratively to mean the destruction of a relationship (burn bridges), or even to refer to emotional pain among many other uses.

To filter out data not relevant to BFA before analysis, we used word embeddings to model the meaning of search terms. Word embeddings represent words as meaningful real-valued vectors of configurable dimensions learned automatically from a large corpus based on their co-occurrence using methods such as fastText [44] or word2vec [45]. We chose to use fastText embeddings because they embed character n-grams. This makes them able to handle some out-of-vocabulary words, which commonly occur on SMPs due to spelling mistakes and typographical errors associated with an informal style of writing. Using the distance in the fastText embeddings space, we extended the original list of search terms (Textbox 1) with 50 words most similar to the BFA search terms (eg, heat, skin, fire, scar, etc) and 50 words most similar to the negated search terms (eg, heartburn, acid, nappy, spicy, etc).

We then proceeded to compare each post against the extended list of search terms. We used an open-source library Gensim [46] to estimate word similarities from their embeddings. Pairwise similarities were calculated between the words contained in a post against the lists of relevant and irrelevant words, respectively. The scores were then averaged to obtain similarity scores for the entire post. In total, 2 thresholds were set empirically for each SMP to classify the corresponding posts as either relevant or irrelevant. Effectively, if a post had high similarity with positive search terms but low similarity with negative search terms, the post was selected as relevant and retained for further analysis.

### Topic Modeling

Topic modeling uses statistical analysis to uncover abstract topics discussed in a collection of text documents [47]. Each topic is associated with a set of words that are extracted automatically from the corpus based on their distribution. The words can help interpret the underlying semantics of each topic. In this study, topic modeling served 2 purposes. First, it was used to filter out irrelevant content in bulk by simply inspecting the words describing the topics and removing all irrelevant topics together with the corresponding documents. Second, it was used to identify the topics in BFA conversations, and the words used to describe them, which can be used to inform the search phase in any subsequent BFA studies.

Topic modeling was performed separately for each SMP, including Mumsnet, Netmums, YouTube, and Twitter. As a result of these experiments, we fixed the total number of topics to 11 across all sets.

Each topic model was evaluated qualitatively following the protocol set out by Spasic and Button [48]. To aid its interpretation, each topic model was visualized using an interactive web-based representation [49]. For more detail on topic modeling methods, see Multimedia Appendix 2.

### Conversations Through Time

The number of posts, comments, and tweets were broken down by calendar year to gain an understanding of the time frame for each data set and any chronological trends in data volume.

### Identifying Parent Voices

Additional opportunities for identifying parent voices in SMP conversations were presented within the Twitter user description and YouTube channel description fields. The extent to which parent voices could be positively confirmed in extracted data was investigated using keyword searches of these fields based on parent descriptor terms as identified in our initial parent involvement survey. As it was not possible to search these user description fields and conversation content concurrently in the same query, this additional data search was necessarily conducted following data extraction.

### Conversation Characteristics

BFA-relevant conversations on each platform were characterized by the proportion of tweets, retweets, and replies on Twitter and the range and median number of followers per user. User-defined location was manually translated to country level (by VB), and the language setting of the tweet was also recorded.

The length of conversations in terms of number of comments (YouTube) or posts in a thread (Twitter, Mumsnet, and Netmums) and number of users participating were summarized (by mean number and range).

Initial posts on parenting forums, YouTube videos (judged using title and description data), and tweets were manually categorized (by VB) as to whether they were asking for advice, offering advice, sharing experiences, or broadcasting news. The initial posts on parenting forums were also further categorized as to the type of burn described and who had sustained the injury (adult, child, or pet).

The top 50 most frequently occurring words across tweets, YouTube comments, and parenting forum posts were listed for each platform, excluding common stop words (using the *tidytext* package in R; R Foundation for Statistical Computing [50]) and pronouns, and investigated for potentially important terms not included in our specific first aid and food terms list defined in our search strategy. The top 10 specific first aid and food terms by the proportion of posts, comments, and tweets they occurred in were then compared between platforms.

## Results

### Search and Filtering

**Figure 1:**
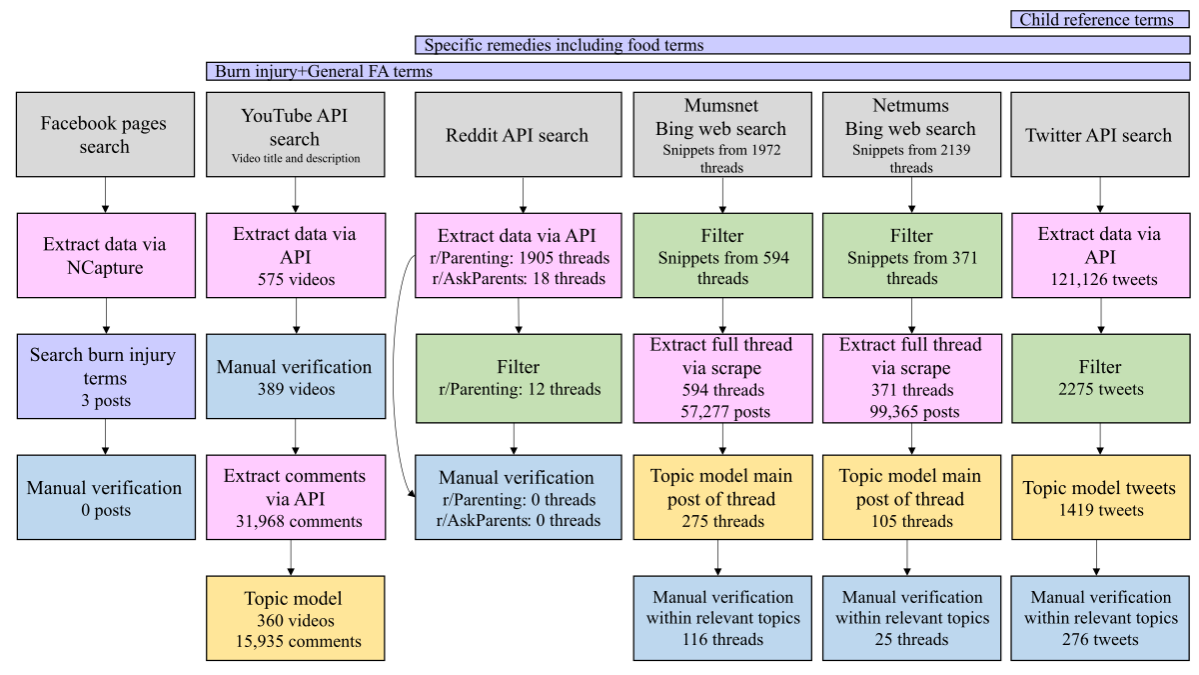
Data flow diagram outlining the search, extraction, and processing approach for each platform. Purple bars at the top of the figure indicate the groups of search terms included in the strategy (refer to Textbox 1 for a list of terms in each group). Child reference terms were only used when searching Twitter data, whereas specific remedies were not included in the search terms for Facebook and YouTube. No search terms were used to extract Facebook data. Grey boxes indicate the platform and data connection or search method. Pink boxes indicate data extraction. Green boxes indicate the filtering step. Purple indicates search based on specific terms. Blue boxes indicate manual (human) verification steps. Yellow boxes indicate topic modeling. Numbers throughout represent the volume of data at each step of the process. API: application programming interface; FA: first aid.

The amount of conversation data resulting from our search and filter methods varied greatly between platforms (Figure 1).

It was possible to extract data from Facebook organization and influencer pages; however, no posts were found on influencer pages following our search for BFA-relevant terms. Only 3 posts were found on the organization pages that contained BFA terms, all of these were signposting to first aid courses and had no specific detail about BFA practice.

The “r/Parenting” search returned 1905 threads, which were then reduced to 12 (0.63%) threads following filtering. None of these threads were relevant to BFA upon manual verification. The “r/AskParents” search returned 18 threads because of the small volume of data, filtering using word embeddings was not conducted, and only manual verification of posts was conducted, finding no threads relevant to BFA.

The YouTube search returned 575 videos, of which 389 (67.7%) appeared via manual verification to be relevant to BFA based on their title. A total of 31,968 comments were then extracted from these 389 videos, and these comments were used in our topic modeling investigation.

Initial searches using Bing returned 1972 and 2139 threads for Mumsnet and Netmums, respectively. Filtered search results reduced the number of threads by 70% for Mumsnet to 594 threads, with a total of 57,277 posts. Filtering reduced the number of threads by 83% for Netmums to 371 threads with 99,365 posts.

The Twitter API search returned 121,126 tweets, which were reduced by 98% to 2275 tweets after filtering.

### Topic Modeling

A small number of automatically extracted topics were confidently interpreted by both reviewers as likely relevant to BFA (highlighted yellow in Multimedia Appendix 3). This facilitated further qualitative analysis by focusing the attention of manual analysis on conversations that belonged to these topics and discarding less relevant conversations automatically.

In total, 2 topics relevant to BFA were identified within both Mumsnet and YouTube data, one topic was identified in the Netmums data, and 3 topics were identified in the Twitter data. Sunburn was identified as a topic across all platforms.

**Figure 2:**
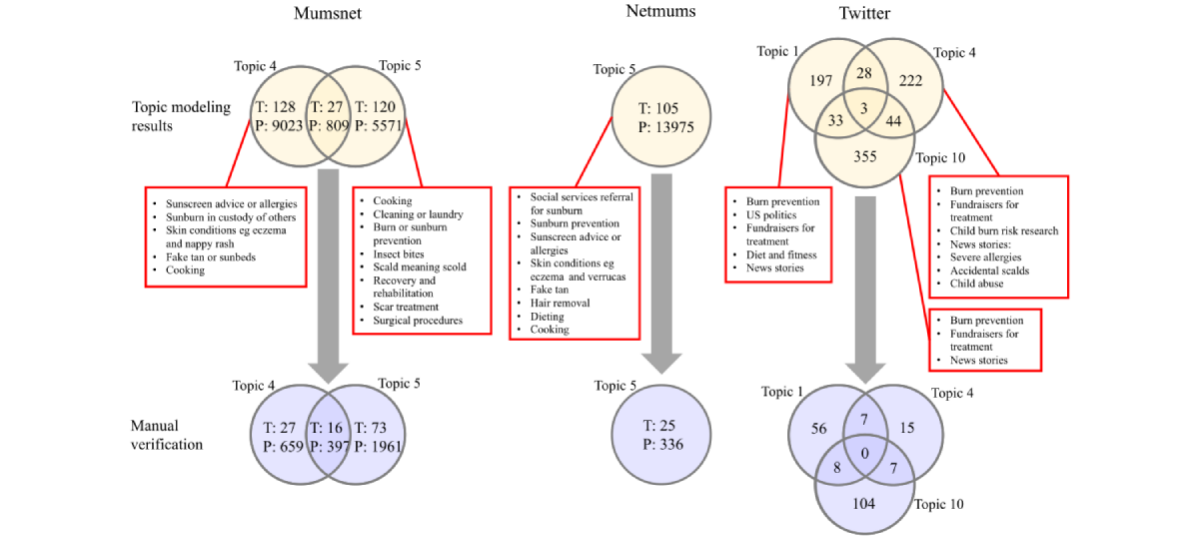
Flowchart to show the process of manual (human) verification of threads and tweets containing information relevant to burn first aid. Circles indicate modeled “topics,” before filtering (yellow circles) and after filtering (blue circles), with the number of threads denoted “T” and the number of participants denoted “P.” Red boxes illustrate the wide range of discussion subjects included in each modeled “topic,” determined by the visual appraisal of conversation text by one of the authors (VB).

The advantage of topic modeling compared to traditional clustering is that topics can overlap, thus reflecting a natural phenomenon that a single discourse may span multiple topics. For Mumsnet, 9.8% (27/275) of threads were assigned to both topic 4 and topic 5, and on Twitter, 22.56% (319/1419) of tweets were assigned to more than 1 topic (Figure 2). Comments from 92.5% (360/389) of these videos were grouped into topics that reviewers agreed were likely to be BFA during topic modeling, and most of these videos (292/360, 81.1%) had comments in both topics 2 and 7.

More than two-thirds (389/575, 67.7%) of YouTube videos were manually verified from their titles as relevant to BFA before topic modeling took place. Conversely, following topic modeling, manual verification of the full text in each thread on Mumsnet and Netmums, and of each tweet in “BFA-relevant” topics identified a substantial volume not relevant to BFA (refer to Figure 2 red boxes). Conversations in topics identified by reviewers as being about sunburn (Mumsnet topic 4, Netmums topic 5, and Twitter topic 10) were more often about burn prevention or recounting instances of sunburn rather than discussing how to treat a burn. Conversations identified as being about scalds on Mumsnet (topic 5) were frequently about cooking, cleaning, or treatment for scars. A large number of tweets were reporting news stories, but burn prevention, US politics, and diet and fitness were also among nonrelevant content.

Manual verification of conversation data identified through topic modeling found 42.2% (116/275) of Mumsnet threads, 23.8% (25/105) of Netmums threads, and 22.3% (197/882) of tweets to be relevant to BFA. Topic overlap remained between topics following manual verification.

### Conversations Over Time

The amount of BFA-relevant conversation data fluctuated considerably over time (Table 1). No BFA-relevant conversation was gathered from Netmums in 2009, 2018, and 2020 or from Twitter in 2011. (The first available data for each platform reflects the platform launch date.) Data collected from YouTube showed a relatively consistent increase toward the present. Considerably more data relevant to BFA was collected from Twitter for 2018, 2019, and 2020 than previous years.

**Table 1:** Number of posts (Mumsnet and Netmums), comments (YouTube), and tweets (Twitter) in the burn first aid–relevant data for each platform over time^a^.

Year	YouTube comments (n=31,968), n (%)	Tweets (n=197), n (%)	Mumsnet posts (n=3017), n (%)	Netmums posts (n=336), n (%)
2004	0 (0)	0 (0)	15 (0.5)	0 (0)
2005	0 (0)	0 (0)	70 (2.32)	0 (0)
2006	0 (0)	0 (0)	144 (4.77)	0 (0)
2007	1 (0)	0 (0)	139 (4.61)	32 (0.1)
2008	38 (0.12)	0 (0)	161 (5.34)	8 (0.02)
2009	151 (0.47)	1 (0.51)	29 (0.96)	0 (0)
2010	227 (0.71)	11 (5.58)	22 (0.73)	39 (0.12)
2011	357 (1.12)	0 (0)	145 (4.81)	35 (0.1)
2012	525 (1.64)	4 (2.03)	52 (1.72)	38 (0.11)
2013	451 (1.41)	11 (5.58)	234 (7.76)	119 (0.35)
2014	368 (1.15)	12 (6.09)	131 (4.34)	23 (0.07)
2015	522 (1.63)	4 (2.03)	168 (5.57)	8 (0.02)
2016	1428 (4.47)	3 (1.52)	112 (3.71)	14 (0.04)
2017	4408 (13.79)	6 (3.05)	313 (10.37)	6 (0.02)
2018	5743 (17.96)	101 (51.27)	310 (10.28)	0 (0)
2019	6893 (21.56)	28 (14.21)	556 (18.43)	14 (0.04)
2020	10,856 (33.96)	16 (8.12)	416 (13.79)	0 (0)

^a^Note that 2020 does not comprise a full year of data as data were collected in June or July 2020.

### Identifying Parents

The 389 YouTube videos manually verified as relevant to BFA belonged to 341 channels, out of which 306 (89.7%) had text in the channel description. Less than one-tenth (25/306, 8.2%) had a parent or child term in the channel description. The use of parent and child terms within the text of the comment was even more unusual at 5.11% (1635/31,968) of comments.

Of the 2275 filtered tweets, 6% (n=138) of the users had a parent term in their user description. Of the 197 tweets manually filtered as relevant to BFA, no users could be identified as parents by using terms in their user description.

### Conversation Characteristics

#### YouTube

Upon analysis of YouTube video descriptions (in addition to titles previously assessed during filtering), 7 (1.8%) of the 389 videos were further assessed to not be relevant to BFA. Across the remaining 382 YouTube videos, the mean number of comments per video was 73.43 (SD 182.28; range 1-1662). At 94.8% (362/382) most videos were offering advice (Table 2), although a small number were news stories (17/382, 4.5%), 2 (0.5%) were narratives of people attempting to treat their own burns, and only 1 (0.3%) user posted a video looking for advice for a burn.

Of those offering advice on YouTube, 8.8% (32/362) referred to a treatment method in the title of the video.

**Table 2:** Number of initial posts (Mumsnet and Netmums), tweets (Twitter), or videos (YouTube), containing relevant burn first aid conversation data according to the purpose of the text (seeking or offering advice, sharing experience, news) for each social media platform.

Purpose of the text	Mumsnet (n=116), n (%)	Netmums (n=25), n (%)	Twitter (n=197), n (%)	YouTube (n=382), n (%)
Seeking advice	112 (96.6)	25 (100)	5 (2.5)	1 (0.3)
Offering advice	0 (0)	0 (0)	126 (64)	362 (94.8)
Experience sharing	4 (3.4)	0 (0)	16 (8.1)	2 (0.5)
News stories	0 (0)	0 (0)	50 (25.4)	17 (4.5)

#### Twitter

Of the 197 tweets relevant to BFA, 44 (22.3%) were retweets (4 retweets as replies). There were 179 users in the data set, and the number of followers for a single user ranged from 0 to 2.7 million with a (median of 532). Almost all tweets (195/197, 99%) were in the English language. Two-thirds (119/179, 66.5%) of users had defined their location as a recognizable country or city. Users were in 20 countries, the most common being the United Kingdom and the United States, each with 31.9% (38/119) of the total number of users.

A large portion of tweets (126/197, 64%; IQR 1480.5) were offering BFA advice (Table 2); either directly giving recommended actions within the tweet text, referring readers to a web page, or a call-to-action advertising a first aid course where the advice would be given. A quarter of tweets (50/197, 25.4%) were news stories, 8.1% (16/197) were tweets from users sharing their experiences, and only 2.5% (5/197) were tweets seeking BFA advice.

Furthermore, 38.9% (49/126) of the tweets offering advice, 6% (3/50) of the news story tweets, 69% (11/16) of the tweets from users sharing their experience, and 100% (5/5) of the tweets seeking advice referred to specific BFA advice with the text of the tweet.

#### Mumsnet

**Figure 3:**
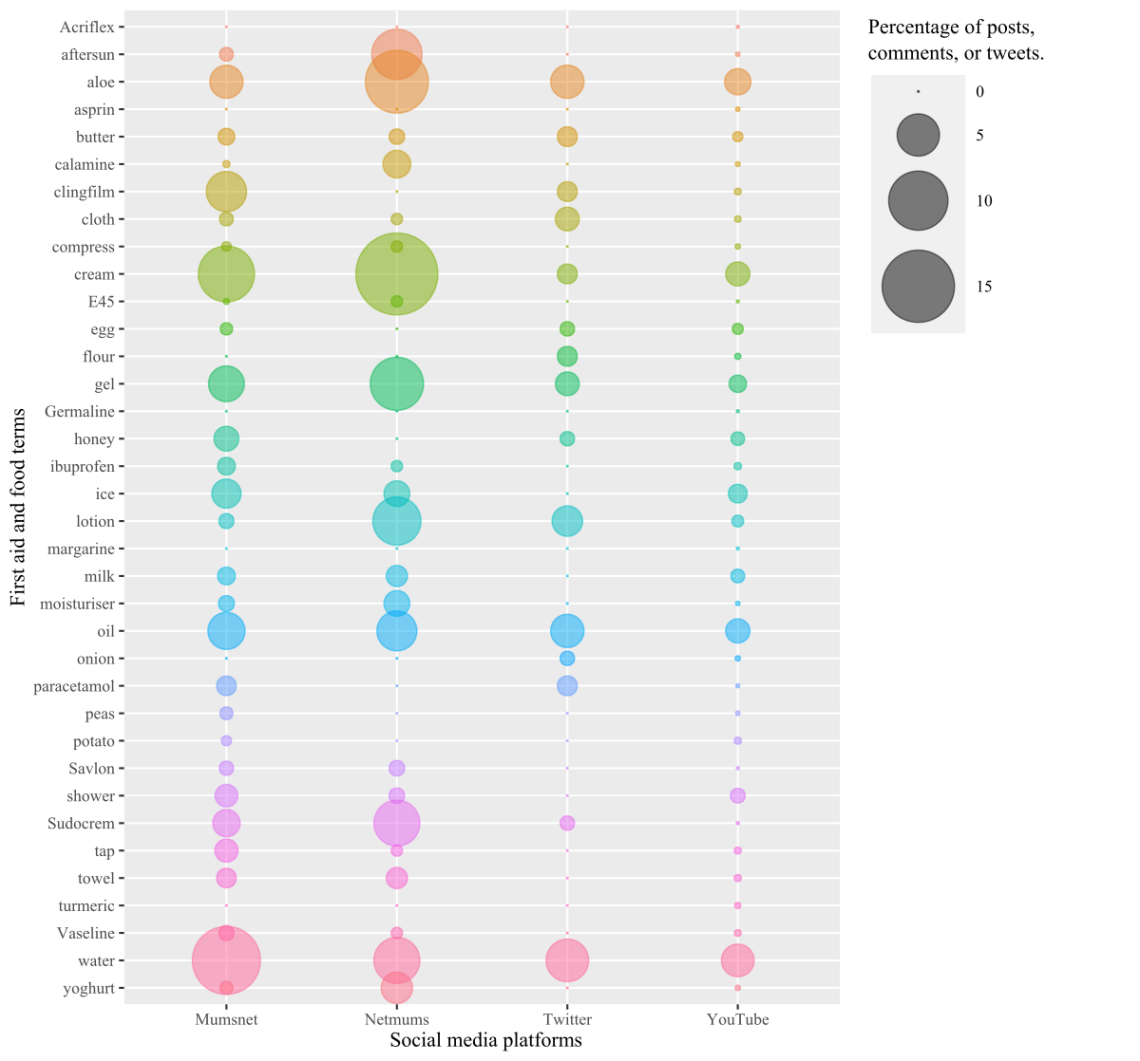
Bubble plot representing the percentage of posts, comments, and tweets containing the specific first aid and food terms from our search strategy for Mumsnet, Netmums, Twitter, and YouTube. The size of the bubble represents the percentage.

Across the 116 BFA-relevant Mumsnet threads, the mean number of posts per thread was 27.5 (SD 36.6; range 2-318). The total number of contributing users across all BFA-relevant threads was 1643, the average number of users in each thread was 15.8 (range 2-233). The first post of most threads was asking for advice (112/116, 96.6%), and a small number (4/116, 3.4%) were sharing their experience of a burn (Figure 3). Of those asking for advice, 91.1% (102/112) were asking for advice on how to treat a burn, a number of which (8/102, 7.8%) were asking for advice following having attended a hospital or clinic for medical attention. Similarly to Netmums, a number of the posts seeking advice (10/112, 8.9%) were asking about a non-BFA related topic, and BFA was introduced to the conversation by other users responding to these posts.

Of the 94 initial posts asking about treating a burn injury, where the person asking had not already received medical advice from a hospital or clinic, 65% (61/94) were asking for a burn sustained by themselves or another adult (16/61, 26% contact burns, 14/61, 23% sunburn, and 12/60, 20% scalds), 32% (30/94) were asking for burns sustained by their child (12/30, 40% contact burns, 9/30, 30% sunburn, (5/30, 17% scalds), 2% (2/94) were asking for burned pets, and 1% (1/94) did not specify who was burned (Table 3).

**Table 3:** Number and percentage of initial Mumsnet posts relevant to burn first aid asking for advice on how to treat a burn injury broken down by burn type and who was injured.

Burn type	Adult (n=61), n (%)	Child (n=30), n (%)	Pet (n=2), n (%)	Not specified (n=1), n (%)	Total (N=94), n (%)
Contact	16 (26)	12 (40)	2 (100)	0 (0)	30 (32)
Sunburn	14 (23)	9 (30)	0 (0)	0 (0)	23 (24)
Scald	12 (20)	5 (17)	0 (0)	0 (0)	17 (18)
Chemical	5 (8)	0 (0)	0 (0)	0 (0)	5 (5)
Steam	4 (7)	0 (0)	0 (0)	0 (0)	4 (4)
Friction	1 (2)	2 (7)	0 (0)	0 (0)	3 (3)
Cold burn	1 (2)	0 (0)	0 (0)	0 (0)	1 (1)
Firework or sparkler	1 (2)	0 (0)	0 (0)	0 (0)	1 (1)
Flame	0 (0)	1 (3)	0 (0)	0 (0)	1 (1)
Laser treatment	1 (2)	0 (0)	0 (0)	0 (0)	1 (1)
Not specified	6 (10)	1 (3)	0 (0)	1 (100)	8 (9)

#### Netmums

Across the 25 BFA-relevant Netmums threads, the mean number of posts per thread was 13 (range 1-84). The total number of users across all BFA-relevant threads was 216 (9 posts had no username given), and the mean number of contributing users in each thread was 9.6 (range 1-42). The first post of every thread (25/25, 100%) was asking for advice (Table 2). Approximately two-thirds (16/25, 64%) of initial posts (were asking for advice about treating burns (14/16, 88% sunburn and 2/16, 13% contact burns), and other posts were initially asking for advice on nonfirst aid topics (9/25, 36%), including sunburn prevention (2/25, 8%), wound care (2/25, 8%), uses for products, for example, Sudocrem (2/25, 8%), after hospital care for a scalded child (1/25, 4%), advice on baby movement after sunbathing while pregnant (1/25, 4%), and what to take on holiday (1/25, 4%). For these posts not initially asking for BFA advice (9/25, 36%), BFA was introduced to the conversation by other users responding to these posts.

Of the 14 initial posts seeking advice for treating sunburns, 7 (50%) users were looking to treat themselves or another adult, and 7 (50%) users were looking for advice to treat a child. Both the posts asking for advice on how to treat a contact burn were referring to a child with a burn.

### Specific First Aid and Food Terms

The percentage of posts, comments, or tweets that contained specific first aid and food terms from our search strategy varied between the platforms (Figure 3). Water, cream, aloe vera (or simply “aloe”), oil, and gel were in the top 10 most frequently appearing of these terms with BFA-relevant conversations on all platforms. These 5 terms were also in the top 50 most frequent words overall (excluding common stop words) for each platform (Multimedia Appendix 4), except for within the Twitter data where oil, gel, and aloe were not as frequently used, and gel was also not as frequently used with the YouTube data.

## Discussion

### Principal Findings

This study is, to the best of our knowledge, the first of its kind to explore the feasibility of extracting large volumes of web-based BFA conversation data. We have designed a suite of bespoke search strategies and composed a catalog of highly relevant keywords and phrases, tailored to a range of different SMPs for the extraction and analysis of these data. Unlike most existing research into health topics on social media, our data were not readily accessible via a specific hashtag or a specific search term. Hence, this methodology will provide a helpful template for others researching other health topics that face similar challenges in semantic characterization.

In alignment with previous research into parents’ use of social media, based on American [51] and Australian [52] data, the most popular SMP determined through our parent involvement work was Facebook. The Pew Research Center [51] reported that 74% of parents on the web used Facebook (81% of mothers and 66% of fathers) and that Facebook parents visit the site more frequently (75% daily and 51% several times a day) than nonparent users (67% daily and 42% several times a day), Facebook showed great potential as a source of health conversation. However, despite extensive use of this SMP by parents, we found that Facebook is of limited facility in big data research into parents’ BFA conversations. This is because much of Facebook’s user-generated content comprises private or participant-restricted conversations, either with friends and family or by joining local parenting support groups. These conversations, on profiles, groups, or direct messages, were not feasible to access from a big data research perspective as it would not be ethical (or indeed possible using the NVivo—Facebook API connection) to access this information without specific individual or group consent.

### Platform Accessibility

Some conversation data were available from Facebook’s public-facing “pages” set up and managed by influencer parents and organizations (as identified by our parent survey). However, once the data were accessed and subjected to our keyword search, we found no relevant BFA conversation on any of the parenting influencer sites and only a small amount of BFA-relevant text consisting of adverts for first aid training posted by the organization itself (eg, St John Ambulance) posted information for BFA around bonfire night in the United Kingdom, and these adverts engendered no opinions or discussion of BFA by those interacting with the page.

Further to the accessibility limitations posed by Facebook, several other SMPs including WhatsApp, Snapchat, TikTok, Pinterest, Ravelry, and Instagram were found to not be feasible for big data research. These SMPs were identified by parents in our initial scoping work, but as they host either entirely private conversations or require an account sign in to read information, we did not investigate accessing these platforms for ethical reasons relating to privacy. This may bear some relevance for the use of SMPs by millennial parents, as although Facebook and YouTube are the most popular SMPs among adults in general, Instagram, Snapchat, and TikTok are more popular with adults aged <30 years [53].

Mumsnet and Netmums were feasible to access by web scrape, and we had confirmation from the site administrators that this action, for this research, should be considered within what could be reasonably expected by their site users. However, “Babycentre,” another parenting website with a similar forum structure, was identified by our parent survey as a potentially relevant source of parent conversations; however, we were unable to make successful contact with the website administrators and so did not explore this platform in our study. It was also feasible to access web-based conversations on BFA and associated metadata from other SMPs, including Twitter and YouTube.

### Finding BFA Conversations

Acquiring a corpus of conversation data with high fidelity to the topic of BFA was challenging due to the low specificity of the keywords associated with burns. For example, the word “burn” appeared to be used widely across SMPs to describe a wide range of various skin discomfort (allergies, rashes, and a general description of pain), or burn terms and common first aid remedies were used metaphorically to refer to embarrassment, insult, or emotional distress. We were able to apply word embedding and topic modeling methods to filter data and home in on more relevant conversations to enable a more manageable data set to then explore manually.

### Identifying Parent Voice

Although 6% of the prefiltered corpus of tweets could be classified as parents using the information in their user description, no BFA-relevant tweets could be classified as such. Equally, YouTube video channel descriptions rarely used parent or child terms. Hence, sufficient parent-generated content to inform interventions is unlikely to be garnered from these sites. Furthermore, identification as a parent could include parents whose offspring are indeed adults, and parents may also choose not to include this in their user description; hence, the utility of the user or channel description to identify parents is severely limited. Considering the nature of BFA-relevant conversation on these platforms, offering advice, signposting to courses and websites, or broadcasting news stories, it would stand to reason that few posts indeed represent parents talking to other parents. Content of BFA advice on YouTube has been previously discussed elsewhere [26] with concerning findings detailing a plethora of inappropriate home remedies for burns. The relative frequency of specific BFA terms and food items compared to “water” on YouTube comments aligns with this finding.

As parenting forums were set up for the primary purpose of parent-specific discussions, that is, Mumsnet describes itself as being “for discussion between parents of children and teenagers” and Netmums describes its web forum as “for mothers to chat, make friends, and exchange advice online,” it is reasonable to assume that those posting on Mumsnet and Netmums platforms are parents or carers of nonadult children and presents a more reliable source of social media conversation between those who would be the target audience of a BFA intervention to improve outcomes for children specifically.

### Implications for Intervention Design and Evaluation

Although we were able to collect a substantial amount of BFA-relevant conversation from Mumsnet, for example, these data were spread across a period of >14 years. Hence, if looking to use these data to measure the change in BFA advice sought or given by parents on this platform before and after an intervention, baseline fluctuation in the amount of data through time, and the number of years of follow-up data collection necessary to compare a sufficient sample size would be important considerations. If looking to use YouTube data for a before and after comparison, we would advise data collection at both the before and after time points separately. A single retrospective search may be biased toward returning more results for the after intervention period due to the increasing trend in relevant posts through time.

Twitter content was mostly offering advice by signposting and broadcasting news, and although there was evident interaction between users retweeting content, there was little evidence of replies to posts, and hence, a lack of interactive discussion to be dissected. Given the lack of positive identification of parents within these data, this means that Twitter holds little utility, at least in the case of BFA, for understanding why parents take certain actions and hence how to change their behavior, unlike Mumsnet and Netmums, which potentially represent a rich source of qualitative data. However, this is not to say that the Twitter data we were able to extract are not without utility. The global distribution of users of Twitter already posting about BFA could help extend the reach of tweets during a web-based campaign. For example, by identifying those with a vested interest in BFA information sharing, these users could potentially be tagged in future campaigns and arguably may be more likely to retweet campaign content.

### Conversation Content

Sunburn was identified as a “topic” at the filtering stage across all 4 platforms. Certainly, there was plenty of conversation around sunburn on both Mumsnet and Netmums, with 24% (23/94) and 88% (14/16) of initial posts, respectively, asking for advice for this type of burn; however, contact burns were also asked about in approximately one-third (28/94, 30%) of initial posts asking for advice on Mumsnet, yet were not identified as a topic during modeling. This could be explained by the highly specific meaning of the word “sunburn” which likely enabled this topic to be more easily discerned than say from the list of keywords when interpreting topics.

The relative proportion of children’s burns for which advice was being sought on parenting platforms differed from those commonly seen in the ED. However, the relative abundance of sunburn posts in our sample might reflect bias due to easier identification as relevant to BFA at the filtering stage mentioned earlier. Bennett et al [14] found 47.4% of attendances for children’s burns at a UK ED to be for scalds, 43.4% for contact burns, and only 4% for sunburn injuries, whereas this study found parents on Mumsnet asked for advice for treating children with contact burns (12/30, 40% of posts) and sunburn (9/30, 30% of posts) more frequently than scald injuries (5/30, 17%). This disparity in results is to be expected as scalds tend to be more serious injuries requiring emergency medical treatment, whereas sunburn is often not as severe and more likely to either be seen in the community or treated at home. However, sunburn in children can be a serious injury, especially if the surface area of the burn is large, and the number of parents seeking advice on how to treat sunburn on parenting platforms might indicate that there exists less clarity and understanding of the best treatment and potential seriousness of such injuries. For Netmums, where the vast majority of initial posts were seeking advice for sunburn, the relatively lower frequency of the term “water” across full threads (20/336, 6% of posts on Netmums vs 400/3017, 13.3% on Mumsnet) and the relatively higher frequency of the term “aloe” (39/336, 11.3% of posts on Netmums vs 91/3017, 3% of posts on Mumsnet) may indicate that parents are less inclined to treat a sunburn with cool running water than the use of aloe vera. The reasons for this remain unclear.

### Limitations

SMPs are continually changing, and the censorship of social media, especially around medical issues, is increasing. In the future, this may remove access to potentially harmful BFA advice. However, this, in turn, may limit our insight into the everyday practices of concerned parents, thus not allowing us to harness the sheer quantity of relevant data that could be used to inform the design of BFA interventions. This study did not evaluate the content of YouTube videos or any links to external sites; hence, our analysis is limited to the text we were able to directly extract from these sites. Future studies may focus on extracting text from the videos either as readily available closed captions or by automatically translating speech to text.

### Conclusions

The potential for social media conversation data to inform the design and evaluation of an intervention to improve parents’ first aid for burns was found, showing both promise and limitations across several aspects of intervention design and evaluation.

Although it was feasible to access BFA conversation data from Twitter, YouTube, Netmums, and Mumsnet, other potentially rich sources of conversation data, such as Facebook parenting groups, could not be accessed. Identification of parents within conversations on Twitter and YouTube via user and channel descriptions was also not fruitful. However, accounts of those already posting about BFA on Twitter and YouTube may represent those with a vested interest in BFA communication. These users could potentially be approached or tagged in future campaigns to assist broadcasting of intervention messages via their channels and accounts to increase the reach of a campaign and improve the appropriateness of their future broadcasts of BFA advice.

Mumsnet and Netmums present an opportunity for in-depth qualitative research into the BFA knowledge, attitudes, and behavior of parents, which could influence intervention design. For example, that parents frequently ask for advice on contact burns and sunburn on these platforms would indicate a need for improved communication of appropriate first aid around these types of burns in particular. The use of remedies other than cool running water could be investigated to unpick the logic and reasons for recommending such remedies, and this could be used to develop targeted myth-busting messages to support parents. Furthermore, the volume of data available from these platforms suggests the potential for monitoring change in conversation content following the launch of an intervention to evaluate intervention impact.

## Appendix

Multimedia Appendix 1 Parent social media use survey.

Multimedia Appendix 2 Further details on topic modeling methodology.

Multimedia Appendix 3 Reviewer topic assignment.

Multimedia Appendix 4 Top 50 terms.

## Abbreviations

API: application programming interface
BFA: burn first aid
ED: emergency department
SMP: social media platform

## Data Availability

The data sets generated and analyzed during this study are available from the corresponding author on reasonable request.
