# Supplementary material for: Assessing the Feasibility of Using Parents’ Social Media Conversations to Inform Burn First Aid Interventions: Mixed Methods Study
Source: JMIR Form Res. 2024 Sep 26;8:e48695. doi: 10.2196/48695 (PMC11467599; doi:10.2196/48695)
Supplement: Multimedia Appendix 1 [file formative_v8i1e48695_app1.pdf]

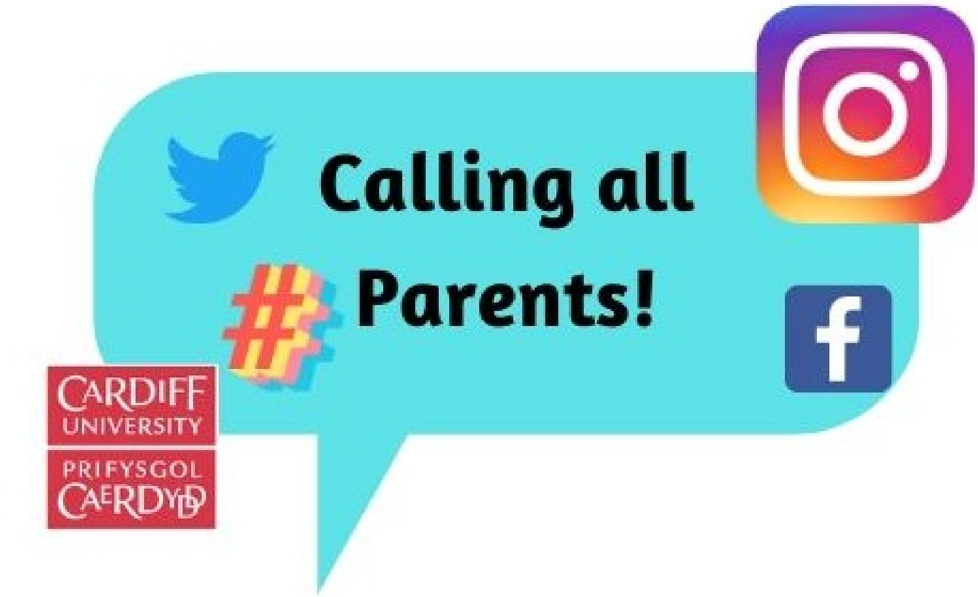

Social Media and Language survey

Page 1: Welcome!

Welcome to the "Burning Questions, Cool Advice" project survey for parents!

50,000 UK children attend hospital emergency departments every year with a burn injury. Correct first aid can lessen the severity, pain and lasting damage caused by a burn. But did you know that only 1 in 4 parents know the correct way to treat a burn?

We increasingly use the internet and social media to find information and advice. This study aims to find out what parents are saying about burns first aid on social media. It will help improve how and where we target campaigns to deliver first aid information for parents. But first, we need your help with where to look and what words to use in our internet search!

Taking part in this survey is entirely optional.

This research is being conducted by researchers at Cardiff University, and funded by The Wellcome Trust. This survey has been approved by the Cardiff University School of Medicine Research Ethics Committee.

All survey data is held securely by Cardiff University via Bristol Online Surveys software. Data will be treated confidentially, accessed only by the project research team and will not be disclosed to any 3rd party organisations. The information you provide will be used solely for academic research or educational purposes.

If you have any questions about the research, please contact Dr Verity Bennett bennettcv@cardiff.ac.uk.

1. I have read and understood the above information about the study and agree to take part in this survey ✱ *Required*

- [ ] Yes

Page 2: Social Platforms

2. Would you ever use these sites for the following reasons? Please check all that apply.

	Asking for advice from parents	Offering advice to parents	Searching for parenting tips	Sharing parenting experiences
	Yes	Yes	Yes	Yes
Twitter	◯	◯	◯	◯
Facebook	◯	◯	◯	◯
YouTube	◯	◯	◯	◯
Instagram	◯	◯	◯	◯
Pinterest	◯	◯	◯	◯
LinkedIn	◯	◯	◯	◯
Snapchat	◯	◯	◯	◯
TikTok	◯	◯	◯	◯
Quora	◯	◯	◯	◯
Mumsnet	◯	◯	◯	◯
Netmums	◯	◯	◯	◯
Gingerbread	◯	◯	◯	◯
Tumblr	◯	◯	◯	◯
Reddit	◯	◯	◯	◯
MySpace	◯	◯	◯	◯
StumbleUpon	◯	◯	◯	◯
CafeMom	◯	◯	◯	◯
Flikr	◯	◯	◯	◯

3. Do you use any OTHER blogs / websites / social media not listed above? If so, please let us know what these are and how you use them below:

4. Have you ever seen first aid / treatment advice for burns on any of these sites?

- [ ] Twitter
- [ ] Facebook
- [ ] YouTube
- [ ] Instagram
- [ ] Pinterest
- [ ] LinkedIn
- [ ] Snapchat
- [ ] TikTok
- [ ] Quora
- [ ] Mumsnet
- [ ] Netmums
- [ ] Gingerbread
- [ ] Tumblr
- [ ] Reddit
- [ ] MySpace
- [ ] StumbleUpon
- [ ] CafeMom
- [ ] Flikr
- [ ] Other

4.a. If you selected Other, please specify:

5. Who are the main influencers / celebrities you follow on social media? please list below:

6. Are you part of any parenting groups on these sites?

- Twitter
- Facebook
- YouTube
- Instagram
- Pinterest
- LinkedIn
- Snapchat
- TikTok
- Quora
- Mumsnet
- Netmums
- Gingerbread
- Tumblr
- Reddit
- MySpace
- StumbleUpon
- CafeMom
- Flikr
- Other

6.a. If you selected Other, please specify:

6.b. If you selected any of the above, please give details.

Page 3: Language

Language on social media can sometimes be very different to how we speak face to face. Abbreviations, acronyms, emojis, slang and regional terms can often make it difficult to understand or search for relevant things that are being said!

Help us out by telling us what other words you might use or ways you might write online to mean the following things:

7. What other words can you think of, or have you seen other people use, when referring to the following online:

	Other words / abbreviations
BURN INJURY e.g. scald, sore, baddie, booboo, mark...	
FIRST AID e.g. treatment, cure, remedy...	
SON e.g. little boy, lad, ds...	
DAUGHTER e.g. little girl, princess, dd...	
CHILD e.g. little one, kid, youngster...	
BABY e.g. infant, tot, newborn...	

PARENTS e.g. Mum, Mummy, Dad, Daddy...	
GRANDPARENTS e.g. Gran, Granny, Grandma, Grandad, Gramps...	

Page 4: Conversations

We're interested in finding out what people write when they search for or give advice.

8. Imagine that your child has touched a hot object and injured themselves. You want to search your social media sites for advice.

Exactly what message/search would you type to find out what to do?

9. Imagine that someone's child has a burn injury and they have asked you what they should do. What would you type in response to that parent?

Page 5: All about you!

We are nearly finished now!

Please let us know a little bit more about yourself so that we can make sure our questionnaire is reaching a wide range of online parents around the country.

10. Your age:

- ◌ under 20
- ◌ 20 - 29
- ◌ 30 - 39
- ◌ 40 - 49
- ◌ 50 or over

11. Are you a:

- ☐ Mum
- ☐ Dad
- ☐ Childminder
- ☐ Aunt
- ☐ Uncle
- ☐ Grandparent
- ☐ Other

11.a. If you selected Other, please specify:

[ ]

12. How many children do you live with / look after?

[ ]

13. What are their ages?

14. What is your nationality?

15. What is your postcode?

16. Before we say goodbye, is there anything that you would like to share with us about using the internet and social media as a parent or to find first aid advice?

17. If you would like to be entered into our prize draw to win a £50 Love2Shop voucher please enter your email address below. (We will only use your email address for the sole purpose of contacting you to arrange delivery of the prize if you win.)

Page 6: Thank you!

If you would like to join our Facebook group to discuss how parents use social media in more detail, please sign up by following the link below:

cardiff.onlinesurveys.ac.uk/parentadvisorygroup

Participants will have another chance to win a £50 Love2Shop voucher, and receive a certificate of participation from Cardiff University.

Thank you for taking time to complete this survey!

Dr Verity Bennett and the Cardiff University Burns Research Team.
