# Supplementary material for: Assessing the Feasibility of Using Parents’ Social Media Conversations to Inform Burn First Aid Interventions: Mixed Methods Study
Source: JMIR Form Res. 2024 Sep 26;8:e48695. doi: 10.2196/48695 (PMC11467599; doi:10.2196/48695)
Supplement: Multimedia Appendix 2 [file formative_v8i1e48695_app2.docx]

### Topic Modelling, Supplementary information

Given that SMP data are normally sparse due to typically short-text nature of the posts, we used a short text topic modelling (STTM) approach [1]. We used an open-source library, which provides implementations of multiple STTM algorithms [2].

Given an STTM algorithm, the performance of a topic model depends not only on the parameters it learned during training (e.g. the weight for each word within a given topic) but also on the hyperparameters (e.g. the number of topics), whose values are fixed manually before the training begins. We ran multiple experiments with different algorithms to measure topic coherence, a measure used to rate topics regarding to their understandability [3].

Each topic was represented by a circle whose size reflected its prevalence in the corpus. The distance between the centres of 2 circles reflected the similarity of the corresponding topics. A histogram of the top 30 words most relevant to a given topic was available on demand by clicking on the corresponding circle.

Two authors with experience in children's burns research, VB and WJW, were presented with an interactive visualization of the topic model. They independently described each topic using a short free-text statement based on the topic's 30 most relevant words. They also estimated their confidence in their interpretation on a 5-point Likert scale: 0 (not confident at all), 1 (slightly confident), 2 (somewhat confident), 3 (moderately confident), and 4 (very confident). In the next phase, both authors gained access to the other one's descriptions of topics. They independently estimated the similarity of the 2 descriptions on a 6-point Likert scale: −3 (very dissimilar), −2 (moderately dissimilar), −1 (slightly dissimilar), 1 (slightly similar), 2 (moderately similar), and 3 (very similar).

Agreement as to the how each of these topics could be described was either judged to be very or moderately similar by reviewers, except for Twitter topic 4, where both reviewers felt the topic assigned by the other was either slightly similar or moderately dissimilar.

References

1. Murshed BAH, Mallappa S, Abawajy J, Saif MAN, Al-Ariki HDE, Abdulwahab HM. Short text topic modelling approaches in the context of big data: taxonomy, survey, and analysis. Artificial intelligence review. 2022:1-128. doi:10.1007/s10462-022-10254-w.

2. Qiang J, Qian Z, Li Y, Yuan Y, Wu X. Short text topic modeling techniques, applications, and performance: a survey. IEEE transactions on knowledge and data engineering. 2020;34(3):1427-45. doi:10.48550/arXiv.1904.07695.

3. Röder M, Both A, Hinneburg A, editors. Exploring the space of topic coherence measures. Proceedings of the eighth ACM international conference on Web search and data mining; 2015.
